# Reversible Cerebral Vasoconstriction Syndrome Due to Midodrine in a Patient with Autonomic Dysreflexia

**DOI:** 10.7759/cureus.4285

**Published:** 2019-03-20

**Authors:** Nidhi Shankar Kikkeri, Elanagan Nagarajan, Keerthivas Premkumar, Premkumar Nattanamai

**Affiliations:** 1 Neurology, University of Missouri, Columbia, USA

**Keywords:** reversible cerebral vasoconstriction syndrome, midodrine, autonomic dysreflexia, multifocal narrowing, diagnostic cerebral angiogram, thunderclap headache

## Abstract

Reversible cerebral vasoconstriction syndrome (RCVS) is a rare neurological condition that typically presents with a sudden-onset thunderclap headache associated with or without focal neurological deficits. The diagnosis is established by the presence of reversible segmental or diffuse cerebral vasoconstriction on diagnostic cerebral angiogram. Autonomic dysreflexia is a known complication resulting from spinal cord injury. It manifests as episodes of flushing, headache, and fluctuations in blood pressure. Midodrine is an alpha-1 agonist that causes vasoconstriction and is commonly used in patients with autonomic dysreflexia. Here, we report the case of a young woman with a history of autonomic dysreflexia, who presented with a thunderclap headache and was subsequently diagnosed with reversible cerebral vasoconstriction syndrome.

## Introduction

Reversible cerebral vasoconstriction syndrome (RCVS) is a rare condition characterized by the sudden onset of severe headaches, along with the diffuse or segmental constriction of cerebral arteries that resolves spontaneously within three months [1]. It can lead to significant morbidity and mortality due to associated complications, including subarachnoid hemorrhage, ischemia, infarction, and hemorrhagic stroke [2]. The majority of the cases of RCVS (in about 63%) are attributed to the use of medications such as selective serotonin reuptake inhibitors (SSRIs), tricyclic antidepressants (TCAs), nasal decongestants, polysubstance abuse, and causes remain unknown in 37% of patients [2]. Here, we report a case of RCVS due to the use of midodrine in the setting of autonomic dysreflexia.

## Case presentation

**Figure 1:**
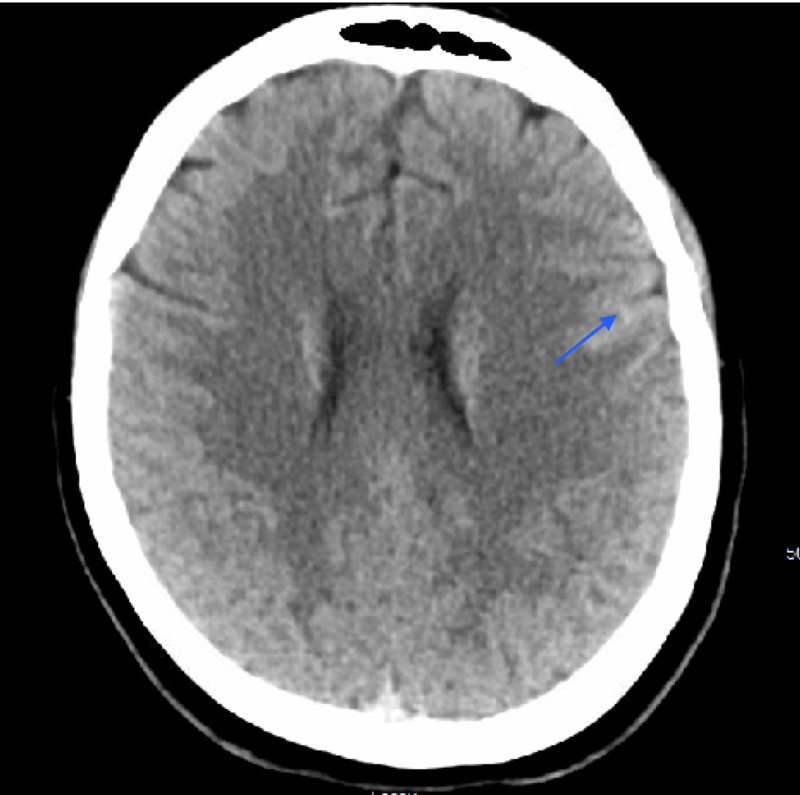
Axial image of CT head showing a hyper-intense signal in the left frontal convexity CT: computed tomography

**Figure 2:**
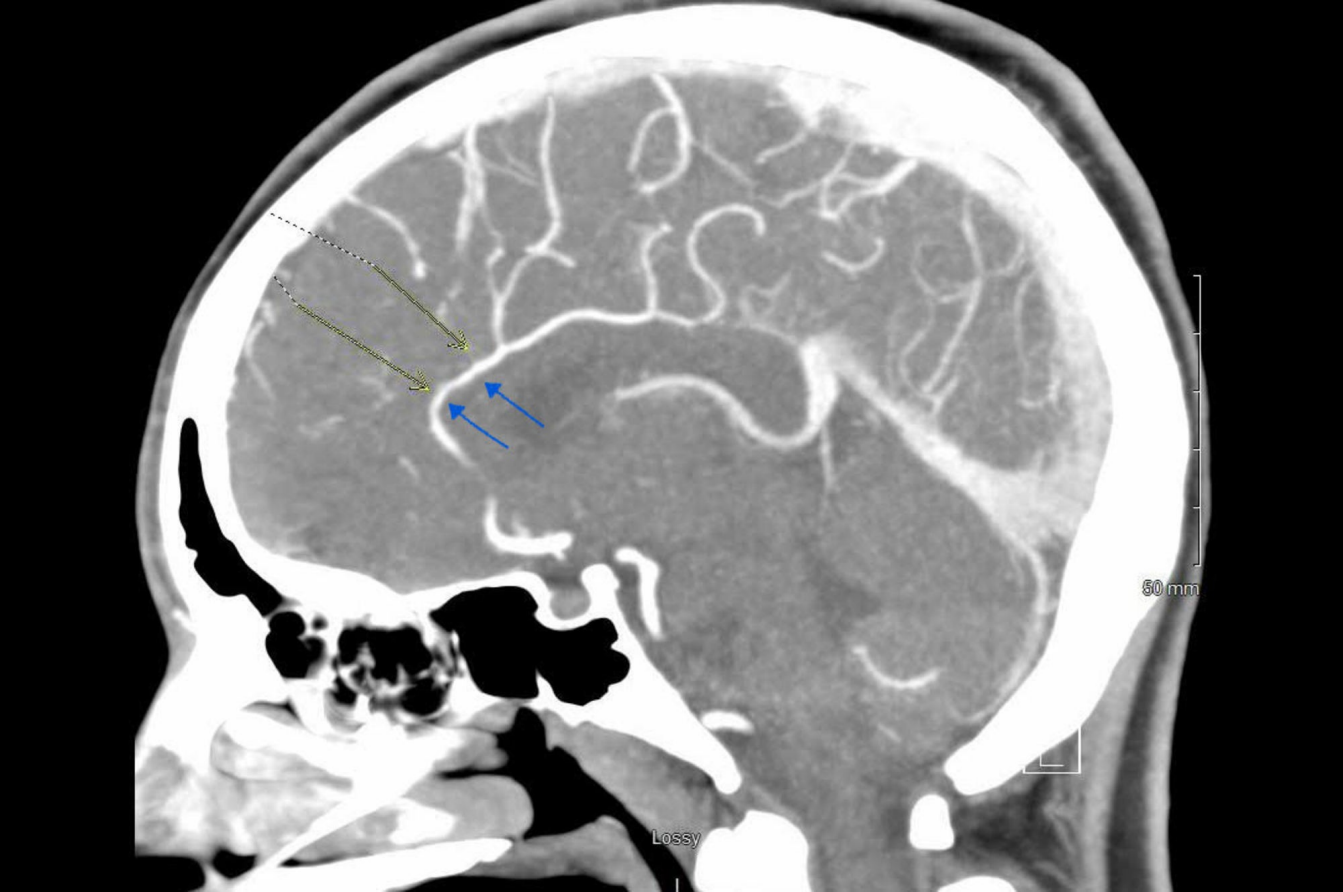
Sagittal section of CT angiogram brain showing the narrowing of the anterior cerebral artery CT: computed tomography

A 23-year-old female presented to the emergency department with a two-day history of recurrent, severe, thunderclap headaches. Headaches were associated with focal motor seizures with secondary generalization. She had a known history of quadriplegia and autonomic dysreflexia from a cervical spinal cord injury. She also reported a blockage of the urinary catheter and experienced recurrent headaches when she attempted to flush the catheter. On our initial assessment, there was no change in the neurological examination from her baseline. Computed tomography (CT) head revealed a subarachnoid hemorrhage as shown in Figure 1. The CT angiogram of the head, as shown in Figure 2, was remarkable for multifocal narrowing of the anterior cerebral artery, bilateral middle cerebral arteries (MCA), right posterior cerebral artery, and pericallosal artery.

**Figure 3:**
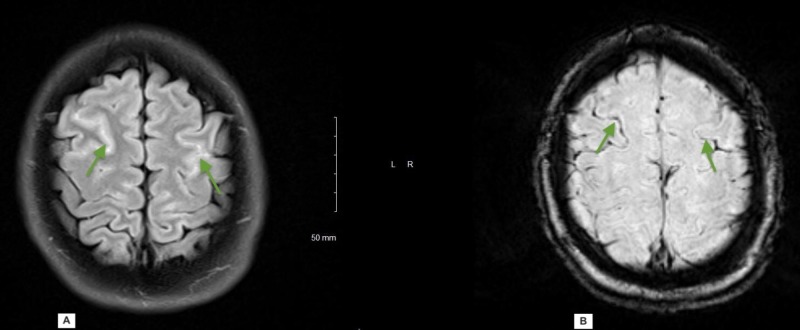
Axial sections of MRI brain T2 FLAIR (A) demonstrating hyper intensity in the bilateral frontal region and prominent blood vessels on the corresponding SWI sequence (B) MRI: magnetic resonance imaging; FLAIR: fluid-attenuated inversion recovery; SWI: susceptibility-weighted imaging

Magnetic resonance imaging (MRI) of the brain, as shown in Figure 3, revealed T2 fluid attenuated inversion recovery (FLAIR) hyperintensity in the bilateral frontal lobes and petechial hemorrhage in susceptibility weighted imaging (SWI). Initial workup, consisting of routine blood tests, erythrocyte sedimentation rate (ESR), comprehensive drug screening, antinuclear antibodies, anti-neutrophil cytoplasmic antibody (ANCA) panel, and angiotensin-converting enzyme, were within normal limits.

**Figure 4:**
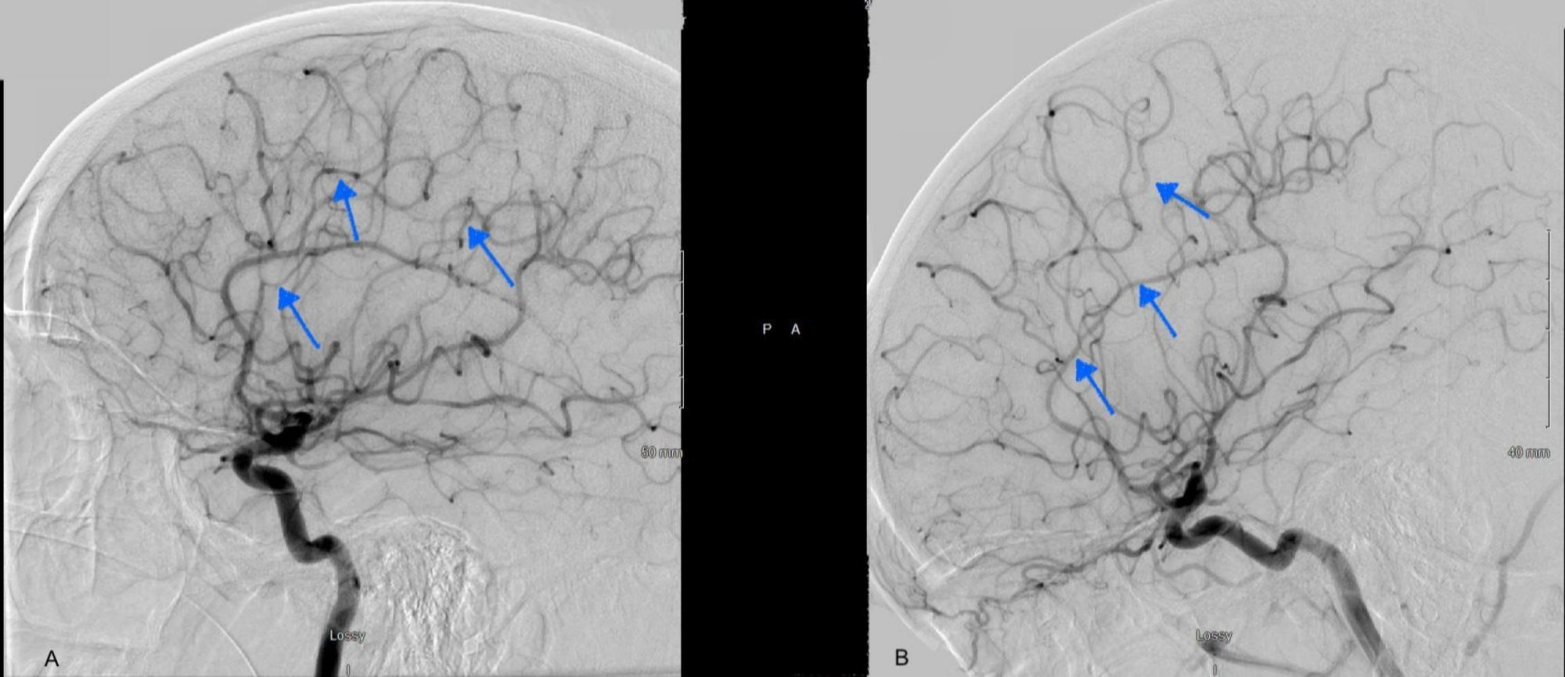
Diagnostic cerebral angiogram at the time of admission showing severe narrowing of the intraluminal vessels, predominantly MCA and ACA distribution (A). Repeat angiogram at three months follow-up (B) showed resolution of the previously seen vasospasm. ACA: anterior cerebral artery; MCA: middle cerebral artery

**Figure 5:**
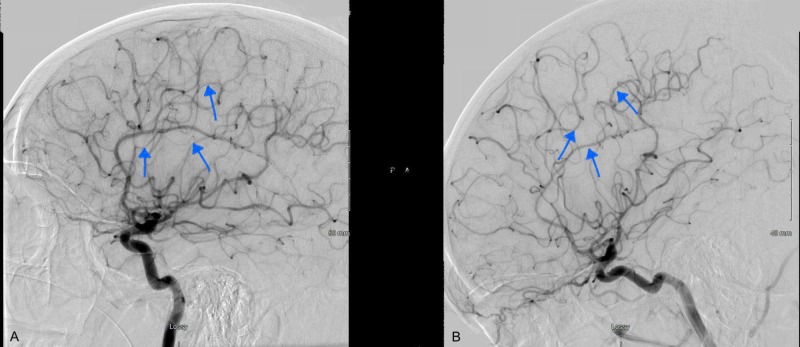
Another view of the diagnostic cerebral angiogram at the time of admission showing severe narrowing of the intraluminal vessels predominantly MCA and ACA distribution (A). Repeat angiogram at three months follow-up (B) showing resolution of previously seen vasospasm. ACA: anterior cerebral artery; MCA: middle cerebral artery

The diagnostic cerebral angiogram was remarkable for stenosis of the bilateral posterior cerebral arteries, callosal and pericallosal branches of the right anterior cerebral artery, and right middle cerebral artery as shown in Figure 4 and Figure 5. During her hospital stay, her headaches recurred, which were associated with episodes of flushing, sweating, and elevated blood pressure. These events were precipitated by unclogging or flushing her foley catheter. Midodrine was discontinued and her headaches improved significantly at the time of discharge. Three months after discharge from the hospital, she underwent repeat diagnostic cerebral angiogram, which was unremarkable as shown in Figure 4 and Figure 5. The patient denied a recurrence of symptoms during the subsequent follow-up visit.

## Discussion

Autonomic dysreflexia is a neurological condition commonly seen in patients with complete or partial spinal cord injury at or below the level of T6 [3]. It is estimated that roughly around 50%-90% of patients with quadriplegia due to spinal cord injury develop an episodic rise in blood pressure, variability in heart rate (tachycardia or bradycardia), anxiety, blurred vision, headache, flushing, and sweating [3-6]. In cases with severe episodic autonomic dysreflexia, patients can have a sudden loss of consciousness, cerebral and spinal subarachnoid hemorrhage, and flash pulmonary edema [7]. These clinical findings are similar to those of our patient, who is a known quadriplegic from a previous spinal cord injury, with episodic autonomic symptoms resulting in headaches and subarachnoid hemorrhage.

It is well-known that autonomic dysreflexia in patients with a spinal cord injury is often precipitated by a noxious stimulus to the bladder, bowel, and skin surface below the level of injury [8]. The noxious stimulus can lead to a massive release of sympathetic mediated noradrenaline and dopamine from the splanchnic vascular bed, which causes severe vasoconstriction, resulting in a sudden rise in blood pressure, skin pallor, and piloerection below the level of the lesion [8]. Our patient experienced similar episodes of flushing, diaphoresis, high blood pressure, and severe headache following bladder distention and manipulation of the Foley catheter with saline flushes.

Roughly around 74% of patients with spinal cord injury are found to have orthostatic hypotension during mobilization and among them, 59% of patients were found to be symptomatic (lightheadedness, dizziness, etc.) [9]. Although the pathophysiology of orthostatic hypotension remains unclear, it is thought to be due to sympathetic dysfunction, alteration in the baroreceptor function, cardiovascular deconditioning, lack of skeletal muscle pumping activity, and electrolyte imbalance [9]. Our patient also had orthostatic hypotension noted during rehabilitation for spinal cord injury and was started on midodrine after she failed the non-pharmacological interventions.

Midodrine is an α-adrenergic agonist that causes peripheral vasoconstriction and is commonly used in patients with orthostatic hypotension after spinal cord injury [9]. Our patient experienced an episodic massive sympathetic response due to noxious stimuli resulting from the bladder distention and manipulation of the Foley catheter. This, along with the use of midodrine, caused vasoconstriction of major cerebral vessels and endothelial damage resulting in RCVS and subarachnoid hemorrhage. The patient's symptoms resolved after the discontinuation of midodrine.

The diagnosis of RCVS is established by the presence of segmental or diffuse narrowing of one or more cerebral arteries and normalization within three months of diagnosis [1]. Subarachnoid hemorrhage can be seen in a patient with stimulus-induced sympathetic dysreflexia, however, vasospasm of intracranial vessels is not commonly associated. In our patient, vasospasm was likely due to the usage of midodrine resulting in subarachnoid hemorrhage. After discontinuing midodrine, the diagnostic cerebral angiogram was repeated at a three-month interval, which did not show any evidence of segmental or diffuse narrowing, thereby confirming the diagnosis of RCVS.

## Conclusions

RCVS should be considered as one of the differentials in patients presenting with a thunderclap headache in the context of the usage of vasoactive agents, serotonergic drugs, or sympathomimetic agents. Caution needs to be exercised while using vasospastic agents, such as midodrine, in patients with autonomic dysreflexia.
